# Electron Paramagnetic Resonance Spectroscopy to Evaluate the Oxidative Stability of Beer, Wine, and Oils

**DOI:** 10.3390/molecules31010041

**Published:** 2025-12-22

**Authors:** Michele Segantini, Angela Fadda, Daniele Sanna

**Affiliations:** 1 Helmholtz-Zentrum Berlin für Materialien und Energie GmbH, Hahn-Meitner-Platz 1, 14109 Berlin, Germany; michele.segantini@helmholtz-berlin.de; 2 Istituto di Scienze delle Produzioni Alimentari, Consiglio Nazionale delle Ricerche, Traversa la Crucca 3, I-07100 Sassari, Italy; 3 Istituto di Chimica Biomolecolare, Consiglio Nazionale delle Ricerche, Traversa la Crucca 3, I-07100 Sassari, Italy

**Keywords:** electron paramagnetic resonance, antioxidant activity, oxidative stability, spin trap, beer, wine, edible oils, benchtop EPR, VCO EPR

## Abstract

Oxidative stability plays an important role in determining the quality of oxidation-sensitive foods and beverages such as beer, wine, and edible oils. Oxidation occurs through radical chain reactions producing off-flavors and leading to deterioration and decrease in the quality and nutritional value of food and beverages. In this context, electron paramagnetic resonance (EPR) spectroscopy has emerged as a powerful and selective technique for investigating reactions involving paramagnetic species, particularly free radicals and transition metal ions. This review provides a critical overview of the applications of EPR spectroscopy in the study of the oxidative stability and antioxidant activity of the above-mentioned matrices. It highlights the main methodological approaches that this technique can offer to gain insight into oxidative processes. Furthermore, current advances in low-cost and portable EPR instrumentation are discussed, along with their implications for broader adoption in both research and industry settings. The aim is to provide an up-to-date literature survey on the application of EPR spectroscopy for studying the oxidative stability and antioxidant activity of beer, wine, and edible oils, providing a methodological tool for academic and food industry researchers interested in monitoring, improving, and extending food shelf life through reliable analytical tools.

## 1. Introduction

Electron paramagnetic resonance (EPR or ESR, electron spin resonance) spectroscopy is the most suitable technique for directly detecting species with unpaired electrons such as free radicals and transition metal ions. In food chemistry, it has emerged as a powerful analytical tool for the determination of antioxidant properties of dietary antioxidants and for the assessment of the oxidative processes affecting the quality and the shelf life of food products [1,2,3,4,5,6]. EPR spectroscopy is a reliable technique for detecting, quantifying, and identifying free radicals, making it particularly useful for studying the oxidative stability of complex food matrices. However, a major limitation of EPR spectroscopy is its inability to directly detect highly reactive radical species like superoxide, hydroxyl, 1-hydroxyethyl radicals or lipid derived radicals [7]. This limit can be overtaken through the use of spin traps and diamagnetic compounds which react with short-lived free radicals, yielding more stable paramagnetic adducts detectable by EPR [7]. The EPR spin trapping technique has been successfully applied to study beer or bulk oils oxidation [8,9,10,11,12]; on the contrary, this analytical approach has been little applied to study wine oxidation, despite the relevance of free radical processes in wine aging and stability [13].

In edible oils, EPR spectroscopy provides insights into oxidation processes by identifying radical intermediates formed during the early stages of lipid peroxidation and the kinetic evolution of radical species when oils are subjected to thermal treatments. In beers, EPR is fundamental to studying the effects of storage conditions and processing techniques on the formation of reactive oxygen species, which impact flavor, aroma, and nutritional value. By offering a highly sensitive method (up to a few nanomole) for tracking oxidative changes, EPR contributes significantly to quality control, product development, and the optimization of preservation strategies in the food industry.

The aim of this review is to provide a critical overview of the application of EPR spectroscopy in the study of the oxidative stability and antioxidant activity of bulk oils, beer, and wine, highlighting the main methodological approaches that this technique can offer to gain insight into oxidative processes. Furthermore, recent advances in low-cost and portable EPR instrumentation are discussed, emphasizing their potential to broaden the accessibility and adoption of this technique in both research and industrial settings.

## 2. Literature Research Review

The scientific community is strongly interested in the oxidation processes of food and beverages due to their impact on product quality and nutritional value. In this context, EPR spectroscopy is an emerging and reliable tool for studying oxidation processes in various food matrices. A literature search of the Scopus database provided useful information on the impact of antioxidants research and the role of EPR spectroscopy in determining oxidative stability and antioxidant activity. Searching for the terms “oxidative stability” or “antioxidant*” yielded 708,013 publications between 1924 and 2026, 190,318 of which were related to “oil*” or “lipid*”, 6805 to wine, and 1256 to beer (accessed on 12 November 2025). The analysis carried out using the “Analyse Results” function of Scopus showed that the number of published papers per year was less than 1000 up to 1988, whereas it increased to 61,464 in 2024. This demonstrates the significant interest of the scientific community in this issue. The country with the largest number of published articles is China (147,415 documents), followed by the United States (84,899 documents), India (78,132 documents), and Italy (33,464 documents). Out of the 708,013 documents found, 4914 are related to “electron spin resonance” or “electron paramagnetic resonance”. According to the literature data, the application of EPR spectroscopy to the study of oxidative stability or antioxidant activity dates back to 1961, when it was used for studying the structure of phenoxy radicals formed by the interaction of benzoyl peroxide and cyclohexyl percarbonate [14]. By that date, the annual trend of publications revealed an overall increase in research output, rising from less than 10 publications in 1987 to 240 in 2025.

The search was further restricted to consider, in the article title, abstract, and keywords, the terms relative to beer, oils. and wine. This approach allowed us to avoid the inclusion of results which discussed these topics only in the introduction of publications. In particular, the search based on the co-occurrence of the terms “oxidative stability” or “antioxidant*” AND “electron spin resonance” OR “electron paramagnetic resonance” AND “oil*” OR “lipid*” resulted in 1432 articles. For beer and wine, the same search yielded a very low number of publications, so it was decided to expand it by deleting the terms “oxidative stability” and “antioxidant*”, obtaining 119 results for beer and 127 for wine.

**Figure 1:**
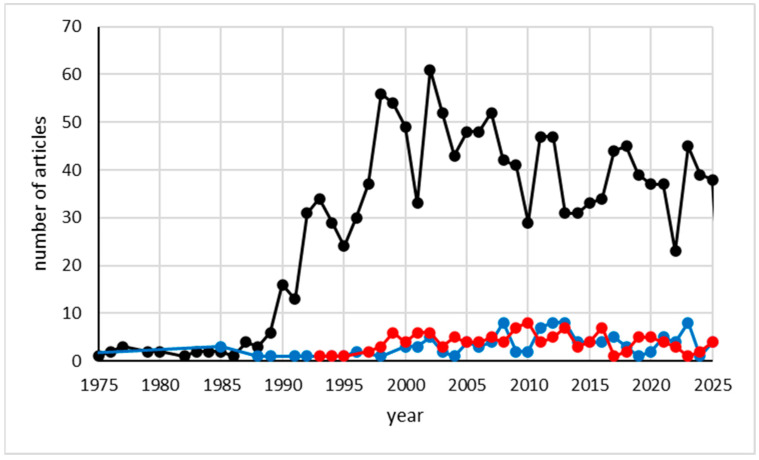
Number of articles published between 1975 and 2025 regarding EPR and oil or lipid (black line), EPR and beer (blue line), and EPR and wine (red line). See text for details.

Figure 1 reports the number of publications per year from 1975 to 2025, for edible oil, beer or wine. The results show the growing interest of the research community in this topic and highlight the impact of EPR spectroscopy in edible oil research. On the contrary, the data analysis shows that the use of EPR spectroscopy is still limited in the beer and wine sectors, despite the relevance of free radical reactions in beer and wine aging.

The outputs related to beer, wine, oil or lipids were saved and imported into VOSviewer software (version 1.6.20, http://www.vosviewer.com, accessed on 24 October 2025) to analyze the relationships among terms used in the titles, keywords, and abstracts, as well as their associated citation data. In analogy with ref. [15], the keywords were selected to ensure the inclusion of pertinent publications in the results. Since EPR spectroscopy is almost exclusively applied to study the antioxidant properties and the oxidative stability of food and beverages, the keywords “antioxidant*” and “oxidative stability” were selected together with electron paramagnetic resonance or electron spin resonance. In this way, we are reasonably sure to include in the results all the studies regarding the oxidative stability and the antioxidant properties of beer, wine, and oils studied by EPR spectroscopy.

**Figure 2:**
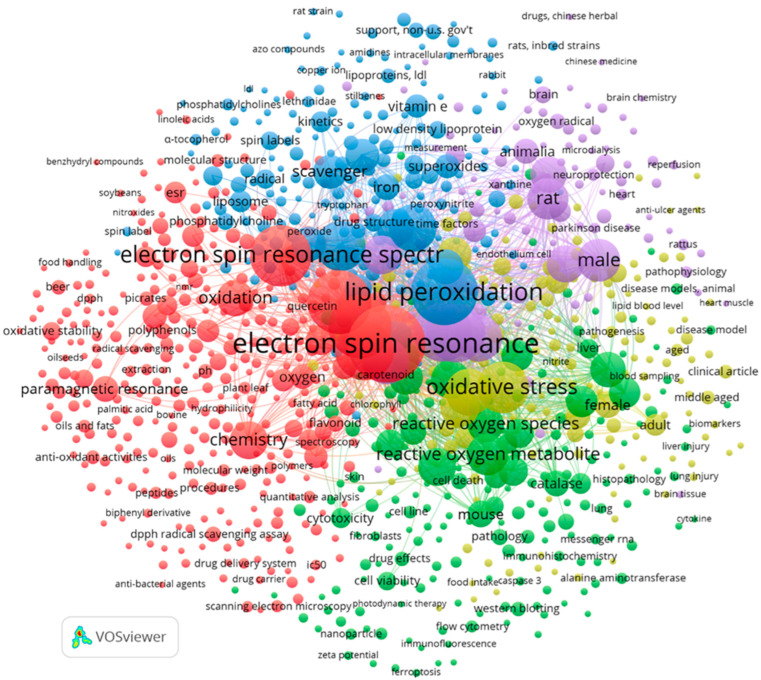
Co-occurrence map of keywords.

The terms were analyzed based on their connections and frequency. Figure 2 reports a co-occurrence map of keywords illustrating the network of connections generated from the references containing the terms “oxidative stability” or “antioxidant*” AND “electron spin resonance” OR “electron paramagnetic resonance” AND “oil*” OR “lipid*” OR beer OR wine, consisting of 1559 records. Before the analysis, the data set was cleaned to remove non-informative keywords and to standardize singular and plural forms of the same keyword.

The keywords were clustered into five groups, each describing a focus area among the imported records. The lines linking different terms show how often a keyword relates to the others, the strength of the links indicates how frequently two terms appear together in the same set of publications, and thicker lines indicate a stronger link, whereas bubble size indicates the number of publications with the specific term.

The red cluster, highlighting the keywords such as beer, wine, lipids, vegetable oils, oxidative stability, polyphenols or lipid oxidation, indicates a network focused on the role of EPR spectroscopy in food chemistry. The relatively small bubble sizes for beer and wine suggest a limited number of publications linking these beverages with EPR spectroscopy. The proximity among terms and the strength of the links indicates how frequently two terms appear together, indicating a strong relationship. For example, the term lipid appears closer to EPR spectroscopy than vegetable oils, suggesting a greater research focus on lipids in general rather than on vegetable oils in the context of EPR studies. The green cluster including the keywords mouse, pathology, animal experiment, cytotoxicity, and metabolism highlights the relationships among antioxidants and cell metabolism, as well as pre-clinical and clinical trials. The cluster with blue bubbles includes keywords dealing with the role of EPR spectroscopy in the detection of radicals or in the assessment of radical scavenging activity. The yellow cluster includes the keywords cardiovascular disease, diabetes, oxidative stress, and humans, highlighting the relationships among oxidative stress and chronic diseases, whereas the violet cluster includes keywords related to the studies on animal models.

## 3. Antioxidant Activity Measured by EPR

In recent decades, there has been a large amount of literature dealing with the free radical scavenging effects of secondary metabolites of food and beverages.

**Figure 3:**
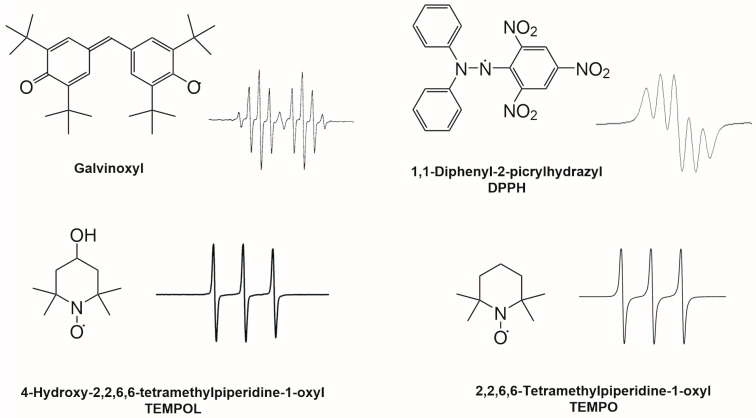
Radicals used in EPR antioxidant activity measurements and corresponding EPR spectra. The spectrum of the galvinoxyl radical can be described as a doublet of quintets due to the interaction of the unpaired electron (*S* = ½) with one H (*I* = ½), the methine group, with a large coupling constant, which gives two lines which are further split into five lines by the interaction with four equivalent aromatic H atoms, giving 2 × *n* × *I* = 5 lines with intensity in the ratio of 1:4:6:4:1. The spectrum of the DPPH radical is described in the text. The coupling constants with the two nitrogen atoms are very similar; the result is a 2 × *n* × *I* + 1 = 5 lines spectrum. The relative intensities of the lines are in the ratio of 1:2:3:2:1. TEMPO and TEMPOL give three lines spectra (2 × *n* × *I* + 1 = 3, where *n* is the number of equivalent nuclei) because of the coupling of the unpaired electron with the ^14^N (*I* = 1).

Several methodologies have been developed to estimate the antioxidant potential of these matrices, based on their capacity to scavenge relatively stable or highly reactive free radicals. EPR spectroscopy is a sound and versatile method to detect free radicals and to estimate antioxidant properties. Antioxidant activity is quantified by the decay of the EPR signal intensity that is proportional to the concentration of free radicals. EPR allows us to detect stable radicals like DPPH, 2,2,6,6-tetramethylpiperidine-1-oxyl (TEMPO), 4-hydroxy-2,2,6,6-tetramethylpiperidin-1-oxyl (TEMPOL) or galvinoxyl radicals and very reactive species like hydroxyl, superoxide, and nitroxide radicals through the spin trapping method [16,17,18,19]. Figure 3 shows the structures and the EPR spectra of some stable radicals. In EPR spectroscopy, stable radicals are often used as internal standards [20] and for the quantification of the antioxidant activity of beverages and food [21,22,23]. TEMPO is a stable nitroxyl radical (Figure 3) successfully employed to study the antioxidant potential of mayonnaise enriched with a grape seed extract [18]. Similarly in beers, TEMPO and TEMPOL (Figure 3) were used to assess, by a kinetic approach, the beers’ reduction power, highlighting the role of SH-containing substances in nitroxide reduction [19]. TEMPOL was also used to show the influence of the addition of ascorbic acid on radical production during thermally initiated beer aging [24].

While TEMPOL and galvinoxyl radicals are mainly detected with EPR spectroscopy, DPPH, being a colored radical, may be detected with UV-Vis spectroscopy. Unlike this methodology, EPR provides some advantages [25,26]. The method is based on the ability of the antioxidant to reduce the DPPH radical to the corresponding hydrazine, thus changing the color of the solution from purple to yellow. The spectrophotometric assay, which is the most widely used, measures the depletion of the DPPH radical in terms of absorbance decay at 517 nm. However, the results in this assay may be affected by some interferences like the absorbance at 517 nm of the colored antioxidants, their oxidation products, or the absorption of the hydrazine when the DPPH radical, initially present, is completely reduced [25,27]. Additionally, the DPPH assay requires sample dilution for dark colors to ensure the correct determination of the absorbance. The EPR assay, relying on radical identification and concentration and not on absorbance, overcomes these pitfalls.

The EPR spectrum of the DPPH radical has two forms. It is a five-line spectrum with hyperfine splitting constants *a*_N1_ = 0.927 mT and *a*_N2_ = 0.846 mT at g = 2.0036 [21] when dissolved in organic solvents such as ethanol or methanol, or in their aqueous solutions, while it shows a single narrow line when in its solid state for high concentrations or when forming aggregates. This enables the EPR spectroscopy assay to identify DPPH aggregation phenomena occurring when high water concentration or high DPPH concentration are used to prepare DPPH working solutions [25]. Some authors applied the EPR spectroscopy to study the DPPH kinetic, identifying slow-, medium-, and fast-reacting antioxidants in lipophilic and hydrophilic extracts and suggesting the role of the flavonoid quercetin as an intermediate-rate antioxidant and anthocyanins as fast-rate antioxidants [21].

In beer samples, EPR spectroscopy in association with the DPPH method was employed to screen 63 commercial beers, demonstrating that the antioxidant properties of beers were affected by the content of the extract and by the color of the beer [28]. Similarly, a total of 182 alcoholic beverage samples, including 62 beers and 62 wines of different origin, were assayed by EPR with the DPPH radical to test a mathematical model able to convert TEAC values into EC_50_ and vice versa [29].

The above-mentioned radicals are synthetic molecules frequently used to provide a fast screening or to give some insight into the antioxidant mechanism when a kinetic approach is applied. In complex systems such as plant extracts or tea samples, rich in different phenolic compounds and metal ions, the DPPH radical scavenging efficiency is determined by the electron and hydrogen donating activity of these compounds. Similarly, the TEMPOL decay is affected by the redox potential of the extracts’ components [30,31].

However, as they are not biological radicals, they provide little information into the antioxidant efficacy of extracts in cellular systems. EPR spectroscopy coupled with the spin trapping method is able to detect highly reactive radicals like hydroxyl (^•^OH) and superoxide (O_2_^•−^), present in biological systems and responsible for many chronic diseases [32,33]. Hydroxyl and superoxide are very short-lived radicals that, after the reaction with a spin trap, a diamagnetic molecule, form a detectable and relatively long-lived paramagnetic adduct. The multiplet structure of the adduct spectrum provides the specific identification of the radical and allows its quantification by the double integration of the spectrum. In food chemistry, the cyclic nitrone 5,5-dimethyl-1-pyrroline N-oxide (DMPO) is the most widely used spin trap for the assessment of the antiradical activity in plant extracts and beverages. While in edible oils and emulsions this spin trap is primarily used to detect lipid radicals generated during oil oxidation, in beer it has been employed to evaluate hydroxyl radical scavenging activity and to correlate it with other parameters related to beer’s oxidative stability and antioxidant capacity [4]. In wine, it is used to study the effect of the chemical composition of wine on the production of hydroxyl radicals driven by the Fenton reaction [34].

**Figure 4:**
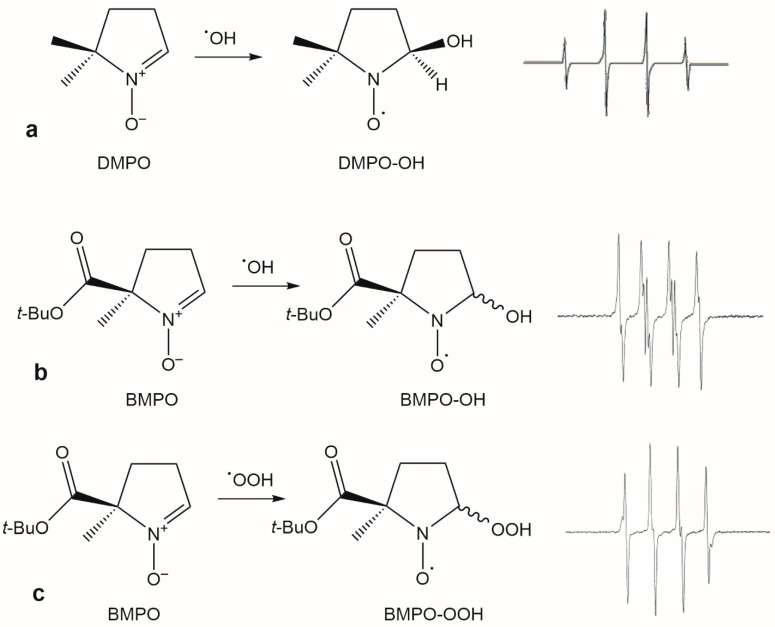
(**a**) DMPO adduct of hydroxyl radical and the corresponding spectrum; BMPO adducts of the hydroxyl (**b**) and superoxide (**c**) radical and the corresponding spectra. The two latter spectra are the sum of two diastereomers; see ref. [39] for details.

DMPO is mainly used to trap hydroxyl radical because, although it can also trap superoxide, the DMPO-OOH adduct undergoes fast decomposition giving the same spectra of the DMPO-OH adduct (Figure 4). In addition, in complex biological systems the DMPO-OH adduct has a limited lifetime, making its detection difficult.

In in vitro spin trapping experiments, experimental conditions significantly impact the results of the antioxidant activity. Factors such as the pH, presence of oxygen, the concentration of the spin trap, and the use of different solvents or buffers may affect the production of hydroxyl radicals through the Fenton reaction and the signal intensity of the adduct [35]. The use of pyridine-2,3-dicarboxylic acid as an Fe(II) chelating agent is an attempt to restrain the effect of pH and the presence of compounds with chelating properties in plant extracts [35,36]. To overcome these limitations, other spin traps like 5-diethoxyphosphoryl-5-methyl-1-pyrroline N-oxide (DEPMPO) or 5-tert-butoxycarbonyl-5-methyl-1-pyrroline N-oxide (BMPO) were developed. DEPMPO is used for in vitro and in vivo analyses, being able to entrap both hydroxyl and superoxide radicals generating adducts with different spectra. Over DMPO, DEPMPO has the advantage to entrap the superoxide radical, obtaining adducts with longer lifetimes. Moreover, the spectrum of the DEPMPO-OH originating from the decay of the DEPMPO-OOH adduct is easily detectable and different from that originating from the reaction of DEPMPO and the hydroxyl radical. These properties make this molecule a reliable spin trap for in vitro detection of ROS in wine and in biological systems [37,38]. Similarly, BMPO (Figure 4) is mainly used to detect superoxide in biological systems [37], but in some works it has been used for the estimation of the antioxidant activity of food or beverages.

## 4. Oxidative Stability Measured by EPR

### 4.1. Oxidative Stability of Beer

EPR spectroscopy has been proposed as an effective technique to predict the shelf life of beer. Starting from the eighties of the last century, some authors used the nitrone spin trap N-tert-butyl-α-phenylnitrone (PBN) to make detectable the highly reactive radicals produced during beer oxidation by converting them into relatively stable paramagnetic PBN adducts and followed the evolution over time of the intensity of their EPR signals [40,41]. The interest in this spectroscopic technique [11,12,42,43,44] led to the publication of a reference method by the American Society of Brewing Chemists [44,45].

This method involves the calculation of the “lag time”, that is, the time of slow growth of the EPR signal intensity, during a forced oxidation test at 60 °C. In this interpretation, the lag time should correlate with the endogenous antioxidant activity of beer. An automated EPR system for the lag time determination in beers was proposed by Bruker [43].

During beer oxidation, the spin trap DMPO identified two radical species corresponding to the spectra of the DMPO adducts of hydroxyl and 1-hydroxyethyl radicals [8]. At a relatively high DMPO concentration (0.5 M), both radicals were detectable. However, when the concentration was reduced to 50 mM, only the 1-hydroxyethyl radical adduct was observed [8]. The 1-hydroxyethyl radical was also trapped by PBN by the same [8] and other authors [46].

Some authors [8] proved that the radical with the highest concentration is the 1-hydroxyethyl radical, but another radical, *tert*-butyl aminoxyl, was also identified [47,48]. This latter radical is formed after acidic hydrolysis of PBN, producing *tert*-butyl hydroxylamine which is further oxidized to *tert*-butyl aminoxyl radical.

Since some authors demonstrated that the determination of the lag time is not always possible [49], the measurement of the lag time was accompanied by other metrics, such as the intensity of the adduct signal after 150 min of incubation at 60 °C (I_150_) [50] and the area under the curve (AUC) representing the intensity of the PBN adducts vs. time [49].

Some attempts have been made to measure the lag time in beer samples, not showing it in normal conditions (in terms of PBN concentration and temperature) by changing the ethanol and PBN concentrations and the temperature at which the samples are heated [4,47]. However, for most of the beer samples the lag time was not measurable, but the shape of the curve representing the intensity of the PBN adduct vs. time changes with the temperature, showed a maximum intensity at shorter time intervals as the temperature increases [4]. Even if in all the examined studies the PBN and final ethanol concentration were not standardized, some attempts have been proposed, such as the addition of PBN in solid form instead of in EtOH solution, to avoid increasing the ethanol concentration of beer [47].

Other parameters have been proposed by other authors, such as the endogenous antioxidative potential (EAP), analogous to lag-time but determined using POBN (α-(4-pyridyl-1-oxide)-N-tert-butylnitrone) instead of PBN, the beverage antioxidative index (BAX), defined as the ratio between the EAP value, expressed in min, and the SO_2_ content of beer in mg/L [51,52]. According to these authors [51,52], PBN has the effect of increasing the pH of the beer, leading to accelerated decomposition of the endogenous antioxidants; this effect is shown to a lesser extent by POBN, since lower concentration of this spin trap can be used due to the higher stability of its adducts [53]. An analogous effect is shown by DMPO: when its aqueous solutions are not acidified, the intensity signals of its hydroxyl radical adduct decrease with increasing its concentration [35].

Recently, the spin trapping method coupled with EPR spectroscopy has also been applied to non-alcoholic beers [48], demonstrating its suitability. In these beers, the limited ethanol concentration led to lower intensity signals of the 1-hydroxyethyl PBN adducts, while the intensity of the *tert*-butyl aminoxyl was comparable in alcoholic and non-alcoholic beers.

However, it is now clear that the lag time is not always measurable, and that two experimental parameters, EPR signal intensity at 150 min or the AUC measured during the same time interval, cannot be used alone because a low I_150_ or AUC could be shown by a completely oxidized beer or by a fresh beer with a high concentration of antioxidants (Table S1). These EPR parameters are significant only in conjunction with others, measuring and quantifying their antioxidant content and their staling degree. Nevertheless, EPR spin trapping methods allow for the rapid and easy determination of beer oxidative status. This would otherwise require the quantification by gas chromatography of molecules indicators of beer flavor deterioration, such as linear and strecker aldehydes, ketones, heterocyclic compounds, and sulfur-containing compounds [54]. Standardized methods of analysis are issued by the American Society of Brewing Chemists (https://www.asbcnet.org/), the European Brewery Convention (https://brewup.eu/), and MEBAK (Mitteleuropäische Brautechnische Analysenkommission, https://www.mebak.org/).

### 4.2. Oxidative Stability of Oils

Recently the EPR spectroscopy coupled with spin trapping techniques has been used to determine the oxidative stability of food lipids, bulk oils, oil emulsions or fried oils by measuring the resistance to the formation of radical species [55,56,57]. Peroxyl and alkoxyl radicals formed during oil oxidation are trapped by PBN, the most widely used open-chain nitrone spin trap, forming a PBN radical adduct detected by EPR. The presence of antioxidants hinders the propagation step of the lipid peroxidation, slowing down the formation of the radical adducts which, being relatively stable, are easily detected by EPR. This technique has been applied to olive, peanut, rapeseed, soybean, sunflower, and fish oils [6,55,58,59,60]. In some of these papers, the oxidative stability measured by EPR was correlated to results obtained with the Rancimat method to propose EPR spin trapping as a precise and easily applicable method for oxidative stability determinations [6,59]. In comparison with the Rancimat method, which measures an induction period, that is, the time until secondary oxidation products are formed in oils exposed to air at elevated temperatures, the EPR method has the advantage of allowing the identification and the quantification of primary oxidation products by trapping them with specific spin trapping agents. Moreover, in these papers, the oxidative stability experiments were carried out by subjecting oils to mild thermal treatments, with temperatures not higher than 70 °C, heating the oils outside the EPR cavity, then measuring the adduct intensity at room temperature [5,55]. Cui et al. [55], for example, stored soybean oil samples for 5 days at 45 °C; samples were incubated for 75 min at 45, 55, or 70 °C before the determination of PBN adduct intensity at room temperature. Velasco et al. [6] heated sunflower and rapeseed oils at 60 °C in a water bath and analyzed the samples for 4.5 h every 30 min. In these papers, a kinetic approach was never attempted since the authors were interested in the formation of the adduct as an indication of the early stages of lipid oxidation. Only in 1995 and later in 1998, in grape seed oil and in fatty acids methyl esters, some authors followed, with the EPR spin trapping technique, the formation and the decomposition of the radical adducts produced during oils thermal treatment, but they did not perform any kinetic analysis [61,62].

Recently, Jiang et al. [60] examined by EPR spectroscopy the formation of PBN radical adducts at 60, 80, and 90 °C in peanut oil, trying to identify an induction period (IP) value by using the procedure suggested by Barr et al. [63].

When an IP value cannot be measured in the curve representing the intensity of the PBN adduct vs. time, other parameters should be used to characterize the oil’s resistance to oxidation. Analogous to the previously proposed method for beer, the AUC in the first 150 min of thermal treatment has been proposed [3,64]. When a maximum is detected in the intensity vs. time curve, this is shifted towards lower time intervals as the temperature is increased [61].

In spin trapping experiments with oils, the extent of their oxidation not only depends on the temperature at which the oils are heated but also on the ratio between the exposed surface of oil and its volume [1,65]. The results, obtained by heating oils in sample holders with large exposed surfaces outside the EPR cavity and then measuring the spectra at RT, are different from those obtained when oils are directly heated in capillary or EPR tubes inside the cavity with very small surfaces exposed to air.

According to the standardized analysis methods for oils outlined in the American Oil Chemists’ Society’s Official Methods and Recommended Practices (https://library.aocs.org/) and the International Olive Council’s Testing Methods (https://www.internationaloliveoil.org/), the oxidative status of oils is described using primary and secondary indicators of oxidation, such as peroxides, *p*-anisidine value, K values, aldehydes, and ketones. These methods analyze the end products of the reactions with lipid radicals but do not provide any indication of the radicals involved. By contrast, EPR spectroscopy coupled with the spin trapping method allows a rapid and straightforward determination of the radical species formed during the initial stages of oil oxidation. These radicals, which are formed by the decomposition of radical precursors during oil storage, first deplete oils’ antioxidants and then generate off-flavor molecules which diminish oil quality.

### 4.3. Oxidative Stability of Wine

EPR spectroscopy coupled with the spin trapping technique has been applied to wine for studying the main radicals involved in its oxidative mechanism.

Using different spin traps, Elias et al. [66] identified three different radical species in wine heated at 55 °C in aerobic conditions. The 1-hydroxyethyl radical was identified as the major radical species and was trapped by POBN; this radical is generated by the hydroxyl radical-mediated oxidation of ethanol. To trap the hydroxyl radical and demonstrate its involvement in ethanol oxidation, DMPO was used. In turn, the hydroxyl radical is generated by Fenton, or Fenton-like, reactions which involve the redox-active metals (iron(II/III) and copper(I/II)) present in wine. The sulfite radicals, originating from bisulfite, were trapped with BMPO.

In wine, the generation of the 1-hydroxyethyl radical has been used to demonstrate antioxidant or pro-oxidant effects of compounds like phenolics and thiols [67].

Other authors measured the intensity of the signal of the POBN–1-hydroxyethyl radical and its rate of formation of white wines and classified them according to these two parameters [13]. Wines with low intensity of the radical adduct and low rate of formation were considered to either hinder the 1-hydroxyethyl radical production or regulate its concentration, indicating a better resistance against oxidation.

## 5. Current Advances in Low-Cost, Benchtop or Portable EPR Instrumentation

### 5.1. Traditional EPR Spectrometers

Electron paramagnetic resonance (EPR) spectroscopy has long been established as a powerful analytical technique for detecting and quantifying free radicals and paramagnetic species in various matrices. EPR has played a crucial role in understanding oxidative deterioration in food systems, particularly in beer and edible oils [68,69].

**Figure 5:**
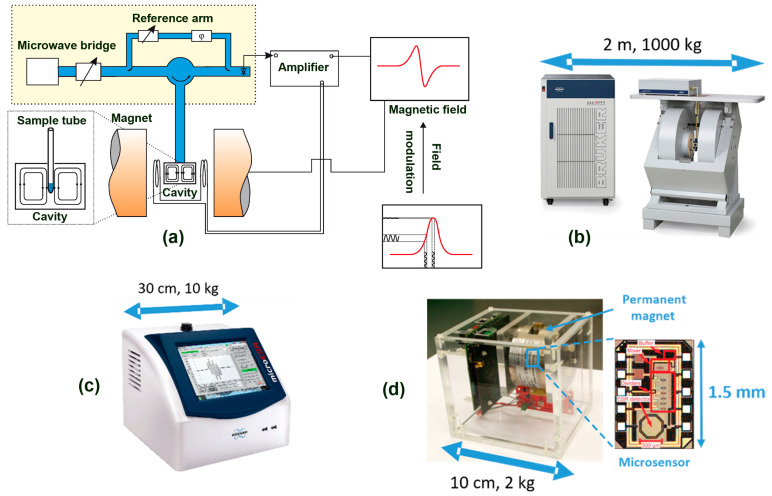
(**a**) Simplified sketch of a conventional X-band continuous-wave EPR spectrometer (reproduced with permission from ref. [73]). Microwave radiation generated by the Gunn diode is directed to the resonator containing the sample; the reflected signal is detected with a lock-in amplifier. As the external magnetic field is swept by the electromagnet and the sample reaches resonance, the EPR spectrum is recorded. (**b**) Image of a traditional X-band EPR spectrometer developed by Bruker. Although these instruments provide state-of-the-art performance, their large size, weight, and high cost restrict their use to highly specialized research laboratories. (**c**) Image of the Micro-ESR spectrometer, designed specifically for beer quality assessment and routine industrial analysis. Picture adapted from https://laballiance.com.my/product/microesr/ (accessed on 25 November 2025). (**d**) Image of a VCO-based EPR sensor, where the instrument dimensions are further minimized through the use of lightweight permanent magnets, enabling customizable designs tailored to specific applications. Additional information on its working principle is available in ref. [74].

Traditional EPR spectrometers employing homodyne detection typically consist of a microwave (MW) bridge containing a MW source and a detector (diode), combined with a microwave resonator (typical volume on the order of a few cm^3^) to enhance the signal-to-noise ratio (SNR). The resonator couples the MW magnetic field component, *B*_1_, to the magnetic susceptibility, χ, of the paramagnetic sample. To record an EPR spectrum, the static magnetic field, *B*_0_, is swept using an electromagnet, while the MW frequency is kept constant. Due to the formation of standing waves inside the resonator, the electric (*E*_1_) and magnetic (*B*_1_) components of the MW field are out of phase, i.e., the maximum *B*_1_ occurs where *E*_1_ is minimal. This configuration is particularly important for samples with high dielectric constant (e.g., aqueous sample such as beer), which can otherwise absorb the MW energy non-resonantly through the electric field component. To further improve the SNR, continuous-wave (CW) EPR spectra are usually recorded using phase-sensitive detection (PSD). With this approach, the static magnetic field is modulated by small modulation coils, and the signal is detected using a lock-in amplifier (LIA). The modulation frequency is typically below 100 kHz, and the modulation amplitude is selected to avoid distortions of the signal linewidth [70]. As a result of this field modulation, the first derivative of the absorption spectrum is detected, as it is shown in the insights of Figure 3 and Figure 4. A concise review of the EPR instrumentation can be found in [71], while a comprehensive treatment is illustrated in [72]. A schematic of the measurement principle of the traditional *X*-band spectrometer is shown in Figure 5a.

As discussed above, the application of EPR spectroscopy to food and beverages quality analysis has provided fundamental insights into oxidative processes, flavor stability, and antioxidant properties. Although highly sensitive and reliable, the conventional EPR spectrometers present significant limitations for routine food quality control, particularly when considering widespread industrial implementation. Indeed, as discussed above, these instruments are typically bulky, costly (often exceeding several hundred thousand dollars), technologically demanding, and non-portable, which limits their use to specialized laboratories. These constraints primarily arise from the large electromagnet and complex control electronics required for operating. An image of a traditional *X*-Band spectrometer is shown in Figure 5b.

### 5.2. Benchtop and Portable EPR Spectrometers

To overcome the limitations of the traditional EPR apparatus, a transformative shift in instrumentation emerged in the early 1980s with the development of specialized benchtop spectrometers. These instruments introduced innovative technical solutions, like the combination of compact electromagnets, permanent magnets, less-complex electronics, and MW sources. An historical description of the development of these instruments can be found in ref. [75]. The reduction in size, cost, and operational complexity has transformed EPR from a specialized analytical technique into a practical tool for routine quality control, allowing food and beverage producers to monitor oxidative processes and product stability on-site without requiring advanced technical expertise. Among the key milestones in the development of tabletop EPR instrumentation is the Magnettech family (MS100, MS200, MS300, MS400, MS5000), which was acquired by Bruker and now commercialized as the ESR5000. This is a benchtop *X*-band system operates over a magnetic field range of up to ~650 mT, with a sensitivity on the order of $\approx 10^{10}\;spins\;mT^{-1}$ and ~50 kg weight. This instrument is highly versatile, supporting the integration of accessories such as a variable-temperature unit (enabling measurements down to liquid nitrogen temperatures), autosampler, optical stimulation module, and continuous-flow cell. Although the ESR5000 is a multipurpose system applicable to a wide variety of materials, it has been extensively used in food and beverage applications. For instance, to study beer stability [76], it is used to investigate the effects of dry hopping on beer flavor stability [77], explore Cu(II) binding in sweet worts [78], evaluate lipid oxidation in shrimp during hot-air drying [79], assess ultrasound-induced off-flavor formation in sunflower oil [80], and determine the antioxidant capacity of olive oil using DPPH [81]. Another of Bruker’s benchtop EPR spectrometers, the e-scan, has been widely employed in beer analysis. Using this spectrometer, a research group from Technische Universität Berlin introduced two EPR-based parameters to assess beer flavor stability: the endogenous antioxidative potential (EAP) and the beverage antioxidant index (BAX) [52]. The EAP parameter enables undistorted quantification of a beer’s flavor stability, while the BAX value provides additional insight into the balance between anti- and pro-oxidative components, independent of SO_2_ content, and reflects the rate of antioxidant depletion during storage. This standardized EPR method has been extensively applied to the analysis of beer and wort [51,82] and can be extended to other beverages such as wine and fruit juices. Following the growing market trend to apply EPR spectroscopy in the food and beverage industry, two additional solutions have been developed specifically for beer analysis. The Micro-ESR, originally commercialized by Active Spectrum Inc. and later acquired by Bruker, overcomes the limitations of conventional ESR spectrometers through the use of a compact rare-earth permanent magnet assembly combined with a low-power (~135 G) electromagnet coil and miniaturized wireless components. The conventional air-core cavity resonator was replaced with a high-Q ceramic resonant cavity possessing a much larger fill factor, thereby maintaining acceptable sensitivity while reducing the overall instrument size by approximately three orders of magnitude to a total weight of only 10.0 kg [83]. For industrial deployment, the Micro-ESR system, offered by FlavorActiV in collaboration with Bruker, integrates automated software and an autosampler for on-site beer freshness and oxidative stability analysis, providing breweries with a complete solution for flavor stability assessment and freshness optimization [84]. An image of the Micro-ESR spectrometer is shown in Figure 5c. Most recently, LineVsystem has introduced application-specific benchtop EPR spectrometers (SpinScan X) optimized for beer analysis and oxidation monitoring, featuring compact X-band magnets, modern digital electronics, and intuitive software interfaces, further advancing the industrial adoption of EPR as a routine quality-control tool [75].

Parallel to the commercial development of benchtop instruments, academic research has driven a second miniaturization frontier through voltage-controlled oscillator (VCO)-based EPR sensors [85]. In these devices, the planar coil of the VCO acts simultaneously as the microwave source and detector, allowing the EPR signal to be read through changes in either oscillation frequency or amplitude. This feature eliminates the need for conventional resonators, microwave bridges, and large electromagnets, enabling handheld spectrometers weighing less than 200 g. A review with the latest advancements in VCO-based EPR devices can be found in [86]. Modern VCO-based sensors can operate in the *S*- (2–4 GHz) [87], *C*- (4–8 GHz) [88], and *X*-bands of (6–15 GHz) [89] and 263 GHz [90] using compact NdFeB or SmCo permanent magnets [74] and achieve spin sensitivities of $6\times 10^{9}\;spins\;\sqrt{Hz^{-1}}$ [91], comparable to commercial benchtop systems. The ability to perform frequency-swept measurements has shown the possibility to create lightweight, compact, and portable EPR sensors integrated with a single-sided permanent magnet to quantitatively detect spins in liquid samples [92]. An image of the first VCO-based spectrometer is shown in Figure 5d. Additionally, submersible VCO-based EPR sensors have been applied to monitor in situ the state of charge in redox flow vanadium batteries [93] through the development of rapid scans [94] at sweep rates up to 14 kT s^−1^; operando oximetry analysis has been conducted in solutions [95]. According to the authors, as stated in ref. [93], future advancements of the spectrometer shown in Figure 5d could find applications in the food industry.

Together with compact benchtop instruments (Magnettech, e-scan, FlavorActiV Micro-ESR™, and LineVsystem SpinScan X), these chip-integrated sensors represent the next stage in the ongoing transformation of EPR technology, from large laboratory instruments to field-deployable, cost-effective analytical tools. The convergence of miniaturized electronics, permanent magnet design, and automated data processing is now positioning EPR as a routine quality-control method for the food and beverage industry, bridging the gap between advanced magnetic resonance spectroscopy and industrial process monitoring.

## 6. Conclusions

EPR spectroscopy has emerged as a powerful and easily applicable tool to detect free radicals and to assess the oxidative stability of food and beverages. The evolution towards miniaturized electronics, permanent magnet design, and automated data processing is making EPR a routine method for quality assurance in the food and beverage sector, linking high-level magnetic resonance spectroscopy to industrial monitoring practices. For EPR spectroscopy to be effectively and efficiently applied in industrial contexts, particularly for food quality control and process monitoring, a thorough understanding of spin trapping experiments is essential. To correctly interpret the spin trapping EPR experiments performed with beers or oils, we should understand what kind of reactions take place during the thermal treatment in the presence of PBN or other spin traps. Should we expect that unsaturated fatty acids react with molecular oxygen to form hydroperoxides which then decompose to form radical species? Or should we expect that redox active metal ions like Fe and Cu react with molecular oxygen to produce hydrogen peroxide, which in turn produce hydroxyl radicals with the Fenton reaction? Considering the low surface of the samples inside the EPR tubes or capillaries, exposed to atmospheric oxygen, and its limited solubility due to high temperatures, it is more likely that precursors of radical species decompose to produce radicals which are trapped by PBN or other spin traps. In the case of beer, these precursors tend to accumulate in the earlier stages of production when oxygen is available, and wort is subjected to heating and cooling steps or can already be present in the raw materials used for the wort preparation. Analogously, precursors of radical species can accumulate in oils after the reaction of unsaturated fatty acids with oxygen during storage. Therefore, during the thermal treatment experiment of beers and oils heated in the presence of spin traps, we should observe the competition between the endogenous antioxidants, present in beer or oil and spin traps for quenching or trapping the highly reactive radicals produced after the thermal decomposition of their precursors. Consequently, the measured values of AUC and I_150_ depend on the ratio between the amount of the radical precursors and the concentration of endogenous antioxidants. Finally, before expressing an opinion about the oxidative status of beers or oils, at least another measurement is needed of its antioxidant capacity or the quantification of primary or secondary oxidation products. This means that the results of spin trapping EPR experiments with oils and beers cannot be taken alone to characterize their oxidative status but should be placed side-by-side with other experimental measurements.

From an instrument point of view, EPR spectroscopy has evolved from a specialized laboratory technique into a practical tool for monitoring oxidation and quality in the food and beverage industry. This transition has been driven by the development of increasingly compact instruments, from benchtop systems such as the ESR5000, e-scan, EMX-nano, Micro-ESR, and LineVsystem platforms to the latest VCO-based devices, demonstrating a clear trend toward portable, low-cost, and application-specific solutions.

While miniaturized systems enable on-site and real-time analysis, they still face limitations in sensitivity, spectral resolution, and experimental flexibility compared to traditional high-performance spectrometers. Future progress will depend on improved resonator and magnet designs, enhanced signal processing, and deeper integration with complementary analytical methods. As these advances converge, EPR is likely to become a standard, routine tool for industrial quality control across the food sector.

## Data Availability

No new data were created or analyzed in this study. Data sharing is not applicable to this article.

## Supplementary Materials

The following supporting information can be downloaded at: https://www.mdpi.com/article/10.3390/molecules31010041/s1, Table S1. Application of EPR spectroscopy coupled with the spin trapping methods for beer analysis. References [4,20,28,47,48,49,77,78,82,96,97,98,99,100,101,102,103,104] are cited in Supplementary materials.
