# Supplementary material for: Electron Paramagnetic Resonance Spectroscopy to Evaluate the Oxidative Stability of Beer, Wine, and Oils
Source: Molecules. 2025 Dec 22;31(1):41. doi: 10.3390/molecules31010041 (PMC12786748; doi:10.3390/molecules31010041)
Supplement: Supplementary file 1 [file molecules-31-00041-s001.zip › molecules-4036095-supplementary.pdf]

Electron paramagnetic Resonance Spectroscopy to evaluate the oxidative stability and the antioxidant activity beer, wine and oils

Michele Segantini[1], Angela Fadda[2,*] and Daniele Sanna[3,*]

1 Helmholtz-Zentrum Berlin für Materialien und Energie GmbH, Hahn-Meitner-Platz 1, 14109 Berlin, Germany
2 Istituto di Scienze delle Produzioni Alimentari, Consiglio Nazionale delle Ricerche, Traversa la Crucca 3, I-07100 Sassari, Italy;
3 Istituto di Chimica Biomolecolare, Consiglio Nazionale delle Ricerche, Traversa la Crucca 3, I-07100 Sassari, Italy;

Supplementary material

Table S1. Application of EPR spectroscopy coupled with the spin trapping methods for beer analysis.

Matrix type	Objectives	EPR experiments	EPR parameters	Main results	References
Non alcoholic beers (NABs)	Measure the lag time in non alcoholic beers	PBN<br>Forced ageing at 60 °C for 150 min	Lag time, I(150)<br>AUC	The I_{150} and the AUC of lagers and NABs were significantly different	[1]
Wort samples	Evaluate the effect of metal chelators on beer oxidative stability.	POBN 7.6 mM (final concentration),<br>Wort forced ageing at 60 °C for 10 hours		Pomegranate extract increased beer oxidative stability (low radical generation) by lowering iron concentration.	[2], [3]
Beer	Evaluate the effect of storage conditions on beer oxidative stability<br>Fresh beer<br>Natural aged beer: 90 days and 180 days at 20 °C<br>Forced aged beers: 7 days and 14 days at 37 °C	PBN 30 mM<br>Forced ageing at 55 °C for 150 minutes	Lag time	Storage conditions affected beer oxidative stability. Increasing storage time and temperatures decreased the lag time.<br>No significant relationship between the concentration of phenolic compounds and ESR-ST values.	[4]

Wort	Evaluate the effect of the incorporation of common solid adjuncts (corn and rice) on wort to increase its flavor stability	PBN 98 mM<br>Forced ageing at 60 °C for 150 min	I(150)	The incorporation of unmalted corn or rice at 25% and 37.5% significantly increased the oxidative stability of sweet worts	[5]
Beer	Evaluate the effect of hop pellets or hop extracts on wort metal ions concentration and on beer flavour stability	POBN<br>Accelerated beer ageing (60 °C) for 500 min	I(500)	The pellet hop addition resulted in a beer with better flavor stability at an equivalent dosage	[6]
Beer	Evaluate the influence of alcohol and PBN concentration on lag time	PBN 50-200 mM<br>Ethanol as is and increased to 9 and 13%	Lag time, AUC and I(150)	Lag time cannot be determined even changing PBN or ethanol concentration	[7]
Beer	Explore how hops with different elemental profiles influence the oxidative stability of dry-hopped beer	(PBN)<br>(0.452 g/mL in 50% v/v ethanol/water<br>Forced ageing at 60 °C for 400 min	I(150) (μM)	Dry-hopping increased the oxidative stability of beer and resulted in the reduction of PBN-adducts measured	[8]
Lager beer	Evaluate the effect of dry hopping at different temperatures: 5 and 20 °C on beers oxidative stability	PBN 30 mM (final concentration)<br>Forced ageing at 60 °C for 140 min	Lag time<br>Radical formation rate	Dry hopping at 20 °C improved oxidative stability of beer better than at 5 °C.<br>Dry hopping at 20 °C prolong lag time of beer<br>The oxidative stability of beer depends on the variety of hops used for dry hopping	[9]
Beer	Evaluate the effect of oxygen scavenging caps on beers stored at 45 °C for 2 and 7 weeks	PBN 50 mM<br>Thermal treatment at 60 °C	Lag time, AUC and I(150)	The highest AUC values are measured after 2 weeks at 45 °C for beers corked with standard and oxygen scavenging caps	[10]
wort	Investigate the Cu(II) binding in sweet worts made from different specialty malts and evaluate the effect of copper concentration on beer flavour stability	POBN 30 microM<br>Forced ageing conditions 60 °C for 90 min	EPR spectra<br>Increase in spin adducts from t_0 and t_{90}	EPR analysis demonstrates in sweet worts an inherent affinity toward binding Cu(II). Accelerated aging experiments with roasted malt worts showed Cu ions may have antioxidative effects in darker worts with high levels of radical formation, while the prooxidative effects may be	[11]

				moderate and only play a significant role in worts with low rates of oxidation.	
beers	Evaluate the influence of yeast strain on the oxidative stability of beer	POBN 168 mg/mL<br>Forced ageing conditions 60 °C for 450 min	lag time<br>I(450)<br>EPR intensity/min (the rate at the point of inflection)	This study showed a significant variation in the oxidative stability of beer produced with different yeast strains.<br>It was not possible to measure the lag time in all beers	[12]
Beer	Detect and quantitate the sulfite (SO_3^-) radical in two different style beers	DMPO 100 mM + PBN 50 mM<br>Forced ageing at 60 °C for 7 hours	EPR spectra	A new C-centered radical was identified	[13]
Lager beers<br>Stout beer	The impact of the transition metal ions on the assessment of oxidative stability	POBN 168 mg/mL<br>Forced ageing at 60 °C for 450 min<br>PBN 452 mg/mL<br>Forced ageing at 60 °C for 150 min	Lag time<br>EPR intensity/min<br>I(450)	The addition of iron to beers lowered their oxidative stability.<br>The addition of manganese ions had a different impact on the tested beers.<br>The addition of copper determined an earlier formation of spin adducts (low lag times) and a low I(450) values	[14]
Beer	Determine the relationship among EPR and spectrophotometric parameters	PBN 50 mM<br>Thermal treatment at 40-80 °C	Lag time, AUC, I(150)	A relationship among EPR and spectrophotometric parameters related to antioxidant activity was found	[15]
Beer	Predict the antioxidant activity with ORAC and FRAP assays in addition to EPR spin trap	POBN 3.5 mM<br>Forcing test at 60 °C	I(150) and I(400), AUC	A combination of ORAC, FRAP and EPR spin trapping is advantageous for the evaluation of antioxidant capacity of beers	[16]
Beer	Find a relationship between EPR parameters and sensory staleness scores for forced aged beers	PBN 100 mM,<br>Beers heated at 27 °C for 18 gays or at 40 °C for 6 days	Lag time, AUC, I(150)	A significant relationship between AUC and sensory staleness scores was found using the 40°C for 6 days, not the 27 °C for 18 days	[17]
Beer	Determination of the antioxidant activity of beer with EPR using the DPPH radical	DPPH 0.2 mM	TEAC mmol trolox/100 ml	The antioxidant activity depends on the extract content and the color, also additives influence but neither the alcohol content or the type of fermentation	[18]

References

1. Porcu, M.C.; Sanna, D. Electron Spin Resonance Spectroscopy Suitability for Investigating the Oxidative Stability of Non-Alcoholic Beers. *Oxygen* **2025**, *5*, 14, https://doi.org/doi:10.3390/oxygen5030014.
2. Mertens, T.; Kunz, T.; Wietstock, P.C.; Methner, F.-J. Complexation of transition metals by chelators added during mashing and impact on beer stability. *Journal of the Institute of Brewing* **2021**, *127*, 345-357, https://onlinelibrary.wiley.com/doi/abs/10.1002/jib.673.
3. Mertens, T.; Kunz, T.; De Rouck, G.; Gibson, B.; Aerts, G.; De Cooman, L. Effects of Mash Chelator Addition on Transition Metal Content and Oxidative Stability of Brewer's Wort. *BrewingScience* **2023**, *76*, 58-72, https://doi.org/10.23763/BrSc23-06mertens.
4. Ferreira, I.M.; Carvalho, D.O.; Guido, L.F. Impact of storage conditions on the oxidative stability of beer. *European Food Research and Technology* **2023**, *249*, 149-156, https://doi.org/10.1007/s00217-022-04140-6.
5. Maia, C.; Cunha, S.; Debyser, W.; Cook, D. Impacts of Adjunct Incorporation on Flavor Stability Metrics at Early Stages of Beer Production. *Journal of the American Society of Brewing Chemists* **2023**, *81*, 54-65, https://doi.org/10.1080/03610470.2021.1993054.
6. Féchir, M.; Dailey, J.; Buffin, B.; Russo, C.J.; Shellhammer, T.H. The Impact of Whirlpool Hop Addition on the Wort Metal Ion Composition and on the Flavor Stability of American Style Pale Ales Using Citra® Hop Extract and Pellets. *Journal of the American Society of Brewing Chemists* **2023**, *81*, 466-479, https://doi.org/10.1080/03610470.2022.2081480.
7. Porcu, M.C.; Fadda, A.; Sanna, D. Lag Time Determinations in Beer Samples. Influence of Alcohol and PBN Concentration in EPR Spin Trapping Experiments. *Oxygen* **2022**, *2*, 605-615, https://doi.org/10.3390/oxygen2040040.
8. Lafontaine, S.; Thomson, D.; Schubert, C.; Müller, I.; Kyle, M.; Biendl, M.; Conn, S.; Schüll, F.; Lutz, A.; Ligare, M.; et al. How deviations in the elemental profile of Humulus lupulus grown throughout the U.S. and Germany influence hop and beer quality. *Food Chemistry* **2022**, *395*, 133543, https://doi.org/10.1016/j.foodchem.2022.133543.
9. Hrabia, O.; Ditrych, M.; Ciosek, A.; Fulara, K.; Andersen, M.L.; Poreda, A. Effect of dry hopping on the oxidative stability of beer. *Food Chemistry* **2022**, *394*, 133480, https://doi.org/10.1016/j.foodchem.2022.133480.
10. Valentoni, A.; Santoru, A.; Sanna, M.; Fanari, M.; Porcu, M.C.; Fadda, A.; Sanna, D.; Pretti, L. Evolution of sensory analysis attributes and volatile aging markers in bottle fermented craft beers during storage at different temperatures. *Food Chemistry Advances* **2022**, *1*, 100151, https://www.sciencedirect.com/science/article/pii/S2772753X22001393.
11. Pagenstecher, M.; Bolat, I.; Bjerrum, M.J.; Andersen, M.L. Copper Binding in Sweet Worts Made from Specialty Malts. *Journal of Agricultural and Food Chemistry* **2021**, *69*, 6613-6622, https://doi.org/10.1021/acs.jafc.1c01582.

12. Jenkins, D.; James, S.; Dehrmann, F.; Smart, K.; Cook, D. The influence of yeast strain on the oxidative stability of beer. *Journal of the Institute of Brewing* **2021**, *127*, 248-255, https://onlinelibrary.wiley.com/doi/abs/10.1002/jib.650.
13. Foster Ii, R.T.; Ranguelova, K. Discovery of Bisulfite and an Uncharacterized Carbon-Centered Radical Systems in Non-Dry-Hopped and Dry-Hopped Beers Using a Different Spin Trap, 5, 5-Dimethyl-1-Pyrroline-N-Oxide, and a New Electron Paramagnetic Resonance Method. *Journal of the American Society of Brewing Chemists* **2021**, *79*, 249-258, https://doi.org/10.1080/03610470.2020.1864699.
14. Jenkins, D.; James, S.; Dehrmann, F.; Smart, K.; Cook, D. Impacts of Copper, Iron, and Manganese Metal Ions on the EPR Assessment of Beer Oxidative Stability. *Journal of the American Society of Brewing Chemists* **2018**, *76*, 50-57, https://doi.org/10.1080/03610470.2017.1402585.
15. Porcu, M.C.; Fadda, A.; Sanna, D. Relationship among EPR oxidative stability and spectrophotometric parameters connected to antioxidant activity in beer samples. *European Food Research and Technology* **2024**, *250*, 2123-2132, https://doi.org/10.1007/s00217-024-04525-9.
16. Rothe, J.; Fischer, R.; Cotterchio, C.; Gastl, M.; Becker, T. Analytical determination of antioxidant capacity of hop-derived compounds in beer using specific rapid assays (ORAC, FRAP) and ESR-spectroscopy. *European Food Research and Technology* **2023**, *249*, 81-93, https://doi.org/10.1007/s00217-022-04135-3.
17. Marques, L.; Espinosa, M.H.; Andrews, W.; Foster, R.T. Advancing Flavor Stability Improvements in Different Beer Types Using Novel Electron Paramagnetic Resonance Area and Forced Beer Aging Methods. *Journal of the American Society of Brewing Chemists* **2017**, *75*, 35-40, https://doi.org/10.1094/ASBCJ-2017-1472-01.
18. Polak, J.; Bartoszek, M.; Stanimirova, I. A study of the antioxidant properties of beers using electron paramagnetic resonance. *Food Chemistry* **2013**, *141*, 3042-3049, https://doi.org/10.1016/j.foodchem.2013.05.133.
